# Supplementary figures and images for: Genome-wide identification and expression analysis of the WRKY genes in sugar beet (Beta vulgaris L.) under alkaline stress
Source: PeerJ. 2019 Oct 14;7:e7817. doi: 10.7717/peerj.7817 (PMC6796966; doi:10.7717/peerj.7817)

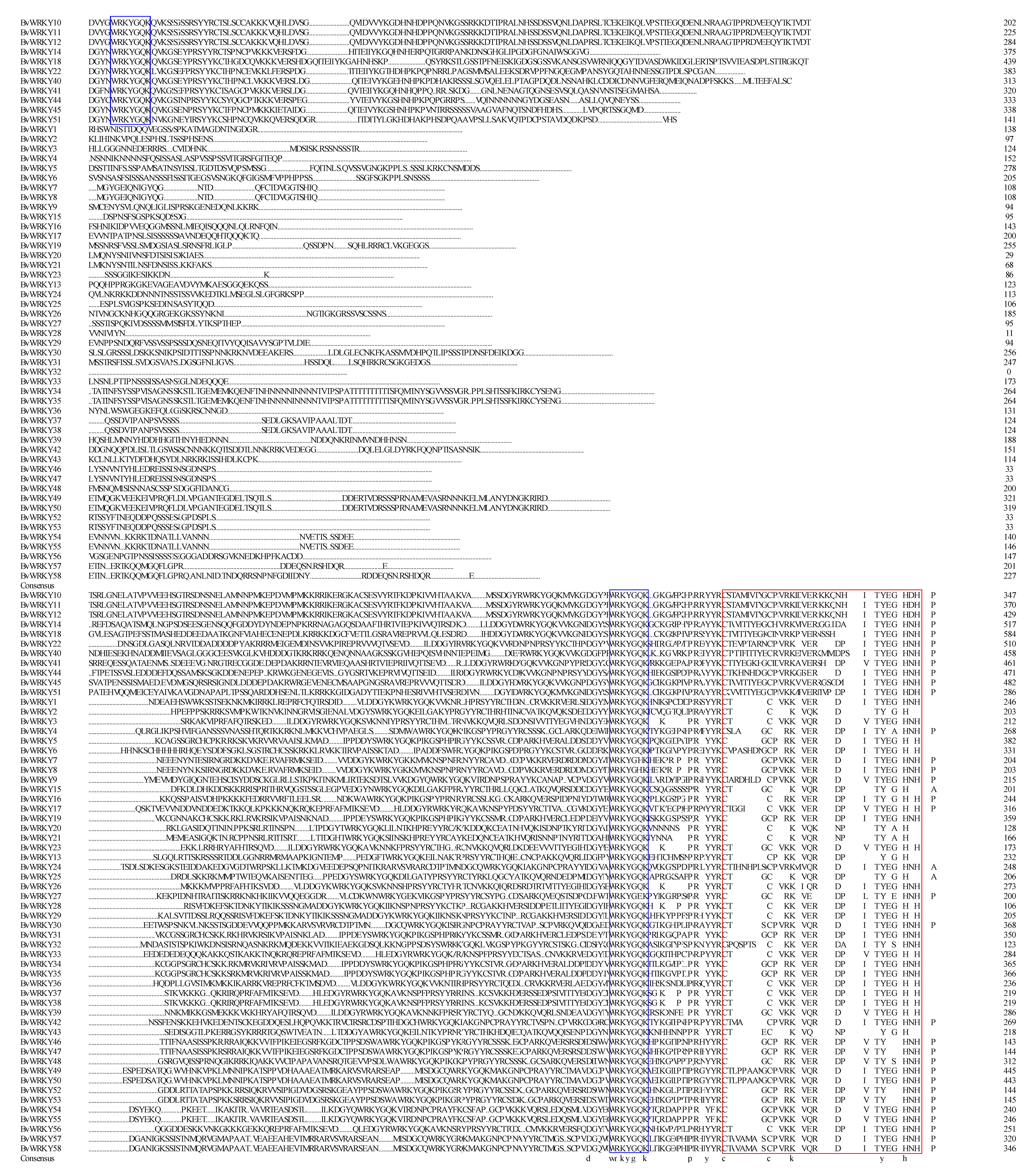

Supplement: Figure S1 [file peerj-07-7817-s002.png]

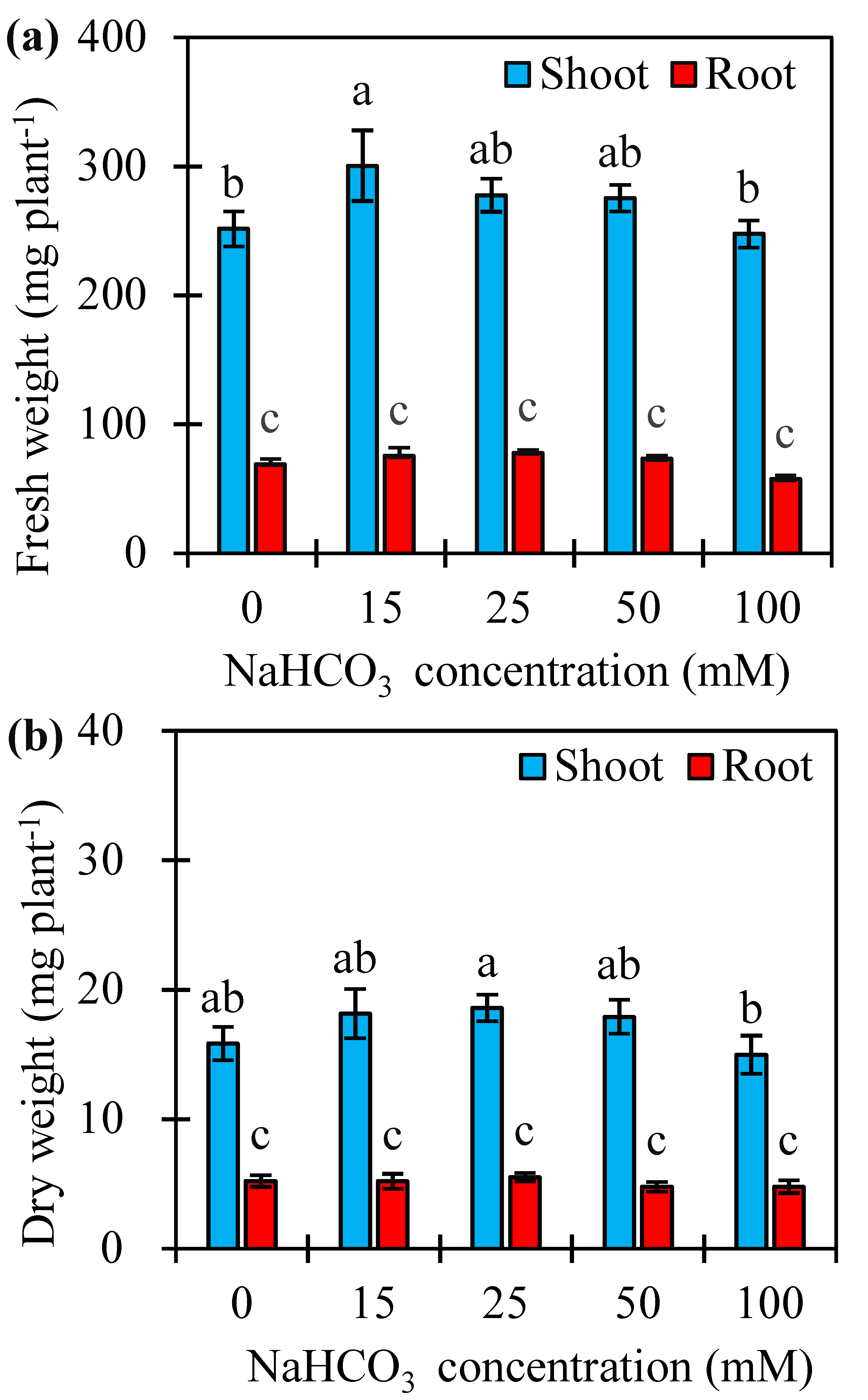

Supplement: Figure S4 [file peerj-07-7817-s005.png]
